# Recon 2.2: from reconstruction to model of human metabolism

**DOI:** 10.1007/s11306-016-1051-4

**Published:** 2016-06-07

**Authors:** Neil Swainston, Kieran Smallbone, Hooman Hefzi, Paul D. Dobson, Judy Brewer, Michael Hanscho, Daniel C. Zielinski, Kok Siong Ang, Natalie J. Gardiner, Jahir M. Gutierrez, Sarantos Kyriakopoulos, Meiyappan Lakshmanan, Shangzhong Li, Joanne K. Liu, Veronica S. Martínez, Camila A. Orellana, Lake-Ee Quek, Alex Thomas, Juergen Zanghellini, Nicole Borth, Dong-Yup Lee, Lars K. Nielsen, Douglas B. Kell, Nathan E. Lewis, Pedro Mendes

**Affiliations:** Manchester Centre for Synthetic Biology of Fine and Speciality Chemicals (SYNBIOCHEM), Manchester Institute of Biotechnology, The University of Manchester, Manchester, M1 7DN UK; Faculty of Life Sciences, The University of Manchester, Manchester, M13 9PL UK; School of Computer Science, The University of Manchester, Manchester, M13 9PL UK; Department of Bioengineering, University of California, San Diego, La Jolla, CA USA; Novo Nordisk Foundation Center for Biosustainability, University of California, San Diego School of Medicine, La Jolla, CA USA; Harvard Extension School, 51 Brattle St., Cambridge, MA 02138 USA; Computer Science and Artificial Intelligence Lab, Massachusetts Institute of Technology, 32 Vassar St., Cambridge, MA 02139 USA; Department of Biotechnology, University of Natural Resources and Life Sciences, Vienna, Austria; Austrian Centre of Industrial Biotechnology, Vienna, Austria; Department of Chemical and Biomolecular Engineering, National University of Singapore, 4 Engineering Drive 4, Singapore, 117585 Singapore; Bioprocessing Technology Institute, Agency for Science, Technology and Research (A*STAR), 20 Biopolis Way, #06-01, Centros, Singapore, 138668 Singapore; Australian Institute for Bioengineering and Nanotechnology (AIBN), The University of Queensland, Corner College and Cooper Roads (Bldg 75), Brisbane, QLD 4072 Australia; Bioinformatics and Systems Biology Program, University of California, San Diego, La Jolla, CA USA; School of Chemistry, The University of Manchester, Manchester, M13 9PL UK; Department of Pediatrics, University of California, San Diego, La Jolla, CA USA; Center for Quantitative Medicine, UConn Health, 263 Farmington Avenue, Farmington, CT 06030-6033 USA

**Keywords:** Human, Metabolism, Modelling, Reconstruction, Model, Systems biology

## Abstract

**Introduction:**

The human genome-scale metabolic reconstruction details all known metabolic reactions occurring in humans, and thereby holds substantial promise for studying complex diseases and phenotypes. Capturing the whole human metabolic reconstruction is an on-going task and since the last community effort generated a consensus reconstruction, several updates have been developed.

**Objectives:**

We report a new consensus version, Recon 2.2, which integrates various alternative versions with significant additional updates. In addition to re-establishing a consensus reconstruction, further key objectives included providing more comprehensive annotation of metabolites and genes, ensuring full mass and charge balance in all reactions, and developing a model that correctly predicts ATP production on a range of carbon sources.

**Methods:**

Recon 2.2 has been developed through a combination of manual curation and automated error checking. Specific and significant manual updates include a respecification of fatty acid metabolism, oxidative phosphorylation and a coupling of the electron transport chain to ATP synthase activity. All metabolites have definitive chemical formulae and charges specified, and these are used to ensure full mass and charge reaction balancing through an automated linear programming approach. Additionally, improved integration with transcriptomics and proteomics data has been facilitated with the updated curation of relationships between genes, proteins and reactions.

**Results:**

Recon 2.2 now represents the most predictive model of human metabolism to date as demonstrated here. Extensive manual curation has increased the reconstruction size to 5324 metabolites, 7785 reactions and 1675 associated genes, which now are mapped to a single standard. The focus upon mass and charge balancing of all reactions, along with better representation of energy generation, has produced a flux model that correctly predicts ATP yield on different carbon sources.

**Conclusion:**

Through these updates we have achieved the most complete and best annotated consensus human metabolic reconstruction available, thereby increasing the ability of this resource to provide novel insights into normal and disease states in human. The model is freely available from the Biomodels database (http://identifiers.org/biomodels.db/MODEL1603150001).

**Electronic supplementary material:**

The online version of this article (doi:10.1007/s11306-016-1051-4) contains supplementary material, which is available to authorized users.

## Introduction

Metabolic processes are implicated in many important aspects of human health. Models are critical in the exploration and understanding of the complexity underlying human metabolism. Genome-scale models of human metabolism [Recon 1 (Duarte et al. 2007) and Recon 2 (Thiele et al. 2013)] have been used to predict biomarkers of inborn errors of metabolism (Shlomi et al. 2009), to identify drug targets (Frezza et al. 2011) and off-target drug effects (Thiele et al. 2013), to study cancer metabolism (Lewis and Abdel-Haleem 2013) and to improve understanding of microbial interactions with the host organism (Bordbar et al. 2010; Heinken and Thiele 2015). Furthermore, while human metabolic reconstructions are of obvious utility in the medical and pharmacological fields, it is worth noting that reconstructions of mammalian biochemical networks also act as blueprints for modelling systems of biotechnological significance, such as Chinese Hamster Ovary (CHO; Kaas et al. 2014) and Human Embryonic Kidney (HEK; Quek et al. 2014) cells. As these networks improve, their applications across basic, clinical and biotechnological research will continue to expand.

The core of a predictive metabolic model is a reconstruction of the underlying reaction network, which catalogues all metabolic reactions encoded within the genome. (One can consider a reconstruction to be a comprehensive knowledge base of biochemistry, covering metabolic reactions and their enzymes, and a metabolic model to be a mathematical representation of the reconstruction.) Naturally, the reconstruction should be both accurate and complete. The most recent human metabolic reconstruction, Recon 2, was published following a large international community effort to develop a consensus reconstruction from existing resources. This reconstruction was of a considerably larger scale than its predecessors and therefore represented an important advancement. Since its publication, Recon 2 has served as a valuable resource for many studies. As is common for such reconstructions, new knowledge about cell metabolism is regularly discovered and these insights must be added to the reconstruction. Furthermore, the use of reconstructions in modelling studies identifies necessary corrections. Thus, for Recon 2, updates and refinements could strengthen the accuracy of its predictions (Swainston et al. 2013; Quek et al. 2014; Kell and Goodacre 2014).

Following the release of Recon 2, several updates were published (Quek et al. 2014; Sahoo et al. 2014, 2015). These updates provided better definition of transport proteins, a wider consideration of drug metabolism, and a number of error corrections. An additional update, Recon 2.1 (Smallbone 2013) focused upon improving carbon balancing but did not cover full stoichiometric mass and charge balancing of every reaction. As such, there is a need for identifying imbalances to ensure accurate predictions of energy metabolism.

In this update, a number of errors are corrected and various improvements introduced to capture human metabolism more accurately and completely. Extensive manual curation has increased the reconstruction size, which now contains 5324 compartmentalised metabolites (of which 2652 are unique chemical species), 7785 reactions, and 1675 associated genes (Table 1). The focus upon mass and charge balancing of all reactions, along with better representation of energy generation, has produced a model that correctly predicts ATP flux on different carbon sources. Thus, through these updates, we have achieved the most complete and best-annotated consensus human metabolic reconstruction available. We demonstrate that Recon 2.2 is, to our knowledge, the first mammalian metabolic model to predict (free) energy production correctly, based upon carbon availability.

The model is freely available from the Biomodels database (Chelliah et al. 2015), under the identifier MODEL1603150001 (http://identifiers.org/biomodels.db/MODEL1603150001).

## Materials and methods

Recon 2.2 is an extension of Recon 2.1 (Smallbone, 2013). A series of manual curation steps led to the development of an interim version, Recon 2.1.5. Following this, a semi-automated curation approach was implemented, in which model updates were specified in simple, human-readable text files. These text files were interpreted by an updated version of the SuBliMinaL Toolbox (Swainston et al. 2011), built on libChEBI (Swainston et al. 2016), in order to automate the production of Recon 2.2 from Recon 2.1.5. All models, text files and software used to build Recon 2.2 are freely available from https://github.com/mcisb/mcisb-recon along with instructions on their use.

Semi-automatic mapping of NCBI identifiers resolved HGNC identifiers but also revealed duplicates, pseudogenes, ESTs and other non-gene records, which have been removed. The resulting reconstruction contains 1675 genes. All gene associations are expressed using disjunctive normal form: (A and B) or (A and C) rather than A and (B or C). This consistency facilitates the writing of parsers, but more importantly is the correct representation of the underlying biology, explicitly specifying genes in terms of the complexes their products form.

Beta-oxidation of fatty acids in the mitochondria and peroxisome was expanded to account explicitly for intermediary fatty acyl-CoA moieties and the full suite of enzymes necessary for complete oxidation of fatty acids. By breaking down lumped beta-oxidation reactions into the component two-carbon cycles, the differing specificities of the enzymes catalysing the first dehydrogenase/oxidase step can be resolved. We additionally clarified the gene-protein-reaction (GPR) relationships for these reactions to include the enzymes required for the full beta-oxidation cycle (acyl-CoA dehydrogenase/oxidase, enoyl CoA hydratase, 3-hydroxyacyl-CoA dehydrogenase, and β-ketothiolase) as well as enzymes utilized for unsaturated fatty acid beta-oxidation (dienoyl-CoA reductase and/or enoyl-CoA isomerase).

The modelling utility of these improvements is demonstrated via a suite of tests. The test suite was developed in Python 2.7, and is available at https://github.com/mcisb/mcisb-recon-analysis. The test suite checks reaction balancing, ATP production under a range of nutrient sources, and the size of the reconstruction and its constituent elements. It can be run against any model that adheres to the COBRA convention (Schellenberger et al. 2011) of SBML formatting, and—similar to the concept of unit testing in software development (Yoo and Harman 2012)—can therefore be used to validate incremental updates to Recon 2.2 as the model develops further.

## Results and discussion

The original goal of Recon 2 was to create a consensus from existing reconstructions. Recently published updates resulted in different versions of Recon 2, and these have all been incorporated into Recon 2.2 to create a new consensus. These include updates to transporter reactions (Sahoo et al. 2014), drug effects and metabolism (Sahoo et al. 2015), and the corrections published by Quek et al. (2014).

Due to their necessity for the development of tissue-specific models, the accurate definition of GPR relationships are of particular importance in multicellular, mammalian reconstructions. GPRs allow the development of models implementing constraints based upon experimentally measured expression data (Lee et al. 2012; Pornputtapong et al. 2015; Uhlén et al. 2015). It follows that the accuracy of such models is directly dependent upon the accuracy of the gene associations in the original reconstruction. Gene association updates from a previous Recon 2 iteration (Recon 2.04, http://vmh.uni.lu) and further manual corrections and updates have also been incorporated into the present version. Previous human reconstructions used a variety of identifiers, mostly from NCBI, to denote genes. In Recon 2.2 genes are now represented using HGNC identifiers, the worldwide authority for assignment of standardised nomenclature to human genes.

A major goal for Recon 2.2 was to improve the simulation of energy metabolism. Towards this end, both mitochondrial and peroxisomal fatty acid oxidation were redefined and expanded by replacing previously lumped reactions with constituent two carbon cycle reactions (e.g. an *n* carbon fatty acid to an *n*-*2* carbon fatty acid), for both saturated and unsaturated fatty acids. Genes associated with fatty acid oxidation have also have been expanded.

Significant improvements also were made by redefining the representation of oxidative phosphorylation. Specifically, this involved the definition of a new compartment, the mitochondrial intramembrane space, and the use of this compartment to define an electrochemical proton gradient. Introducing this specific pool of transmembrane protons enforces the coupling between the reactions of the electron transport chain with that of ATP synthase, and thus the coupling of mitochondrial NADH oxidation and O_2_ reduction with ATP production (Martínez et al. 2014). While there may be inevitable simplifications of the representation of the mitochondrial intramembrane space in the model, its addition greatly improves the results of ATP flux predictions and will act as an incentive for further subsequent curation. The updated reactions are given in Supplementary Information: Table S1.

Recon 2 included reactions with incomplete mass and charge balancing (Table 1). Despite well over 90 % of reactions being correctly balanced, the presence of even a small number of incorrectly balanced reactions is sufficient for leaks (the erroneous or ‘alchemical’ creation of mass) to occur, which can lead to unreliable flux predictions. To address this in Recon 2.2, an automated reaction balancing method, originally introduced in the SuBliMinaL Toolbox software suite (Swainston et al. 2011), was extended and applied. The original algorithm employed linear programming to check and correct reaction stoichiometry based upon element and charge counts of the reaction participants. It was also able to add ‘missing’ protons and water molecules, which are commonly absent from reaction definitions. This algorithm has been extended here to balance reactions involving non-specific metabolites, that is, those containing generic R-groups (‘Markush structures’), or those containing repeating units [e.g. (CH_2_)_n_]. The use of R-groups is especially prevalent in Recon 2 in defining lipid metabolism, where in the interests of simplicity, multiple reactions involving fatty acids of differing chain lengths were condensed into a single reaction. The R-groups that remain are those representing conserved moieties such as acyl-carrier protein (ACP), which cannot be represented as a defined chemical formula but whose presence do not affect the mass and charge balancing of reactions.

Reversible reactions that thermodynamics suggest should be unidirectional under typical physiological conditions can also impact the accuracy of model predictions. Many of these have been discovered through multiple rounds of manual curation, driven by the requirement to predict realistic ATP yields. This iterative process involved performing a flux-balance analysis (FBA) test, inspecting the resulting flux pattern for anomalous reactions, and correcting their directionality based on literature searches and definitions in pathway databases.

Previous work has highlighted the advantages of augmenting metabolic reconstructions with unambiguous, publicly available identifiers mapping elements to entries in persistent, external data resources (Herrgård et al. 2008). Due to its breadth and accuracy, ChEBI (Hastings et al. 2016) has become a de facto standard for the annotation of metabolic species in systems biology models. Recon 2.2 has been further curated to expand the number of metabolites that are annotated to ChEBI entries. Additionally, metabolites that are not currently in the ChEBI database have been submitted to the ChEBI curators with the intention of expanding the database, and incorporating these new ChEBI identifiers into a subsequent iteration . In the interim, Recon 2.2 metabolites have been annotated with InChI string representations of molecular structure.

**Table 1:** Reconstruction scope

	Recon 2.2	Quek	Recon 2.1	Recon 2.04	Recon 2	Recon 1
Metabolites	5324	4962	5056	5063	5063	2766
Reactions	7785	7327	8089	7440	7440	3742
Balanced	7780	6761	7916	6948	6948	431
Unbalanced	0	211	51	94	94	
Unknown	5^a^	355	122	398	398	3311
Genes	1675^b^	2167	2193	2140	2191	

Recon 2.2 has increased the number of metabolites and reactions catalogued in the reconstruction relative to Recon 2, and the reactions have now been verified and corrected for mass balance. Note that boundary metabolites are excluded from this table (boundary metabolites being those outside of the system). The values for metabolites and reactions therefore correspond to the size of the stoichiometry matrix

^a^The 5 unbalanced reactions remaining in Recon 2.2 are those relating to the biomass objective function

^b^There appears to be fewer genes in Recon 2.2 than in previous reconstructions, due to the movement to representing genes by HGNC identifiers, which group alternative transcripts or splice variants into a single entity

A comparison of maximum ATP yields per unit of carbon source was calculated for a number of existing models, under both aerobic and anaerobic conditions. The results show that, in contrast to previous versions, Recon 2.2 is able to correctly predict maximum ATP fluxes. The results are given in Table 2 and Supplementary Information: Table S2.

**Table 2:** Comparison of maximum ATP yields under different carbon sources and oxygenation

Carbon source	ATP yield					
	Theoretical^a^	Recon 2.2	Quek	Recon 2.04	Recon 2	Recon 1
Aerobic						
glc, aerobic	31	32	∞	∞	∞	∞
fru, aerobic	31	32	∞	∞	∞	∞
C4:0, aerobic	21.5	22	∞	∞	∞	∞
C6:0, aerobic	35.25	36	^b^	^b^	^b^	^b^
C8:0, aerobic	49	50	∞	∞	∞	∞
C10:0, aerobic	62.75	64	∞	∞	∞	^b^
C12:0, aerobic	76.5	82.5	∞	∞	∞	^b^
C14:0, aerobic	90.25	92	∞	∞	∞	∞
C16:0, aerobic	104	106.75	∞	∞	∞	∞
C18:0, aerobic	117.75	120.0	∞	∞	∞	∞
C20:0, aerobic	131.5	134	∞	∞	∞	∞
C22:0, aerobic	145.25	147.25	∞	∞	∞	^b^
C24:0, aerobic	159	160.5	∞	∞	∞	∞
C26:0, aerobic	172.75	170.75	∞	∞	∞	∞
Anaerobic						
glc, anaerobic	2	2	∞	∞	∞	∞
fru, anaerobic	2	2	∞	∞	∞	∞
C4:0, anaerobic	0	0	∞	∞	∞	∞
C6:0, anaerobic	0	0	^b^	^b^	^b^	^b^
C8:0, anaerobic	0	0	∞	∞	∞	∞
C10:0, anaerobic	0	0	∞	∞	∞	^b^
C12:0, anaerobic	0	0	∞	∞	∞	^b^
C14:0, anaerobic	0	0	∞	∞	∞	∞
C16:0, anaerobic	0	0	∞	∞	∞	∞
C18:0, anaerobic	0	0	∞	∞	∞	∞
C20:0, anaerobic	0	0	∞	∞	∞	∞
C22:0, anaerobic	0	0	∞	∞	∞	^b^
C24:0, anaerobic	0	0	∞	∞	∞	∞
C26:0, anaerobic	0	0	∞	∞	∞	∞

Glc, glucose; fru, fructose; C*n*:0, a saturated fatty acid of length *n*

^a^Theoretical yields are calculated according to Salway (2003)

^b^Carbon source was not present in the model

To summarise, Recon 2.2 compiles updates from the various different updates to Recon 2.0 that have been published. In addition, hundreds of novel manual updates have been included, and semi-automatic checks have been conducted to create a new consensus human metabolic reconstruction and associated model. Importantly, Recon 2.2 has eliminated all mass leaks from improperly balanced reactions that resulted in previous models being able to simulate growth without a carbon source. ATP synthesis is also now coupled to carbon availability. Simulation of growth and energy metabolism using Recon 2.2 gives biologically realistic results.

Annotations in Recon 2.2 have been improved by increasing the use of ChEBI identifiers for metabolites, and standardising gene annotations to HGNC. During this process numerous misannotations were removed and new annotations incorporated.

The development of Recon 2.2 followed a semi-automated approach. The introduction of this approach provides full traceability of the updates implemented, and will facilitate and accelerate the process of developing subsequent updates (to both the human and any other constraint-based metabolic model). Regarding simplicity, updates are supplied in simple text files that are parsed and interpreted by custom software. By providing the facility to update models in such an automated manner, the potential user base for the model building process expands beyond those that have an intimate understanding of software, and of formats such as SBML and the COBRA Toolbox. The benefits in terms of promoting reproducible science are also clear: all changes made to a given model to produce a new version are catalogued in text files, which essentially act as a *diff*^1^ between versions. Finally, once the underlying software to interpret the text files has been written, the process of further developing future iterations of the model rests solely in writing updated text files. This approach allows the model developer to focus on the *content* of the updates, rather than the technical means of implementing them.

## Electronic supplementary material

Below is the link to the electronic supplementary material.

Supplementary material 1 (XML 23979 kb)

Supplementary material 2 (PDF 18 kb)

## Footnotes

1 From Wikipedia: *In computing, the diff utility is a data comparison tool that calculates and displays the differences between two files.*
