# Supplementary material for: Recon 2.2: from reconstruction to model of human metabolism
Source: Metabolomics. 2016 Jun 7;12:109. doi: 10.1007/s11306-016-1051-4 (PMC4896983; doi:10.1007/s11306-016-1051-4)
Supplement: Supplementary file 2 — Supplementary material 2 (PDF 18 kb) [file 11306_2016_1051_MOESM2_ESM.pdf]

Supplementary Information Table S1: Redefined reactions of oxidative phosphorylation. The fundamental change is in the redefining of the protons as being in the intramembrane space, where previously these were represented simply as cytosolic protons. Changes include those to respiratory chain reactions and the aspartate-glutamate shuttle, all of which involve the transport of intramembrane protons. Abbreviations: q10 (coenzyme Q10), q10h2 (ubiquinol-10), ficytC (ferricytochrome C), focytC (ferrocytochrome C), glu_L (L-glutamate), asp_L (L-aspartate), [m] (mitochondrion), [i] (mitochondrial intramembrane space), [c] (cytoplasm)

**Reaction id**	**Reaction name**	**Reaction definition**
NADH2_u10m	NADH dehydrogenase, mitochondrial	5 h[m] + nadh[m] + q10[m] -> nad[m] + 4 h[i] + q10h2[m]
CYOR_u10m	ubiquinol-6 cytochrome c reductase, Complex III	2 ficytC[m] + 2 h[m] + q10h2[m] -> 2 focytC[m] + 4 h[i] + q10[m]
CYOOm3	cytochrome c oxidase, mitochondrial Complex IV	4 focytC[m] + 8 h[m] + o2[m] -> 4 ficytC[m] + 2 h2o[m] + 4 h[i]
ATPS4m	ATP synthase (four protons for one ATP)	adp[m] + pi[m] + 4 h[i] -> atp[m] + h2o[m] + 3 h[m]
ASPGLUm	aspartate-glutamate mitochondrial shuttle	h[i] + glu_L[c] + asp_L[m] --> h[m] + glu_L[m] + asp_L[c]

Supplementary Information Table S2: Predicted maximum ATP yields on different amino acid carbon sources under aerobic conditions.

	**ATP yield**
**Carbon source**	**Recon 2.2**
Ala	15
Arg	27.5
Asn	15

Asp	15
Cys	17. 5
Gln	22.5
Glu	22.5
Gly	8
His	25.5
Ile	35.5
Leu	33.5
Lys	32
Met	27
Phe	33
Pro	27.5
Ser	13.5
Thr	19
Trp	39.5
Tyr	35.5
Val	29
